# Social capital, depressive symptoms, and perceived quality of care among hypertensive patients in primary care

**DOI:** 10.1186/s12955-020-01630-7

**Published:** 2020-12-01

**Authors:** Haitao Li, Hui Xia, Shijian Yi, Lichang Rao

**Affiliations:** 1 Shenzhen University General Hospital, Shenzhen University Clinical Medical Academy, Xueyuan AVE 1098, Nanshan District, Shenzhen, China; 2 Center for Chronic Diseases Prevention and Control, Longhua District, Shenzhen, China; 3 Hebei Research Institute for Human Resources and Social Security Science, Hebei, China

**Keywords:** Depression, Hypertension, Primary care, Social capital, Quality of care

## Abstract

**Background:**

Depression is an important issue in the management of hypertension. However, little attention has been paid to addressing such aspects of psychological health among patients with hypertension. We aimed to estimate the prevalence of depressive symptoms among patients with hypertension in primary care settings and to identify the potential role of social capital in predicting depressive symptoms. The influence of psychological well-being on the perceived quality of hypertensive care was also examined.

**Methods:**

In Shenzhen, China, an on-site cross-sectional study was conducted from March to September 2017. In total, 1046 respondents completed a face-to-face survey interview. We examined the associations between social capital, depressive symptoms, and perceived quality of care.

**Results:**

The results showed that 10.7% of patients with hypertension who attended primary care facilities had depressive symptoms. Two components of social capital—social ties (9.63 vs. 10.67; OR = 1.314, 95% CI 1.165–1.483; *P* < .001) and trust (3.46 vs. 3.89; OR = 2.535, 95% CI 1.741–3.691; *P* < .001)—were protective factors for depression among patients with hypertension in primary care settings. We also found that depressive symptoms were negatively associated with perceived quality of care (30.5 vs. 32.5; *β* = 1.341, 95% CI 0.463–2.219; *P* = .003)..

**Conclusions:**

We found inverse associations between depressive symptoms and perceived quality of care and between social capital and the occurrence of symptoms of depression. Our findings suggest that strategies addressing both hypertension and depressive symptoms should be implemented to better manage hypertension. Appropriate social interventions should be designed and implemented.

## Background

The incidence of hypertension is high and increasing worldwide, especially in developing countries. Hypertension is considered an important public health challenge. As of 2014, approximately one billion adults—about 22% of the global adult population—had hypertension [1]. Previous studies have predicted that around 1.56 billion adults will suffer from hypertension by 2025 [2]. As is the case in other developing countries, a sharp increase in hypertension has been found in China over the past several decades. In 2010, 34% of Chinese adults aged over 25 years were found to have hypertension [3]. Among cardiovascular diseases, hypertension is the most widespread and has the highest mortality rate, globally. Because of the condition’s widespread nature and high morbidity, research related to hypertension should be highly prioritized.

Recently, researchers have paid substantial attention to the psychological well-being of hypertensive patients. Psychological well-being can be conceptualized as having positive feelings and thoughts toward life. In contrast, psychological distress is broadly defined as a negative interval state of the individual that is independent of the interpretation or appraisal of threat, harm, or demand. Existing studies have indicated that psychological distress and related psychological factors such as depressive symptoms are associated with the progression of hypertension. There is a higher incidence of depression among patients with hypertension than among other patients in primary care (PC) settings, and depressive symptoms increase as hypertension progresses [4]. A study using data obtained from two pulmonary hypertension centers in the United States found evidence of depressive symptoms among 50% of patients with pulmonary hypertension [5]. A study by Mashele et al. [6] reported that depression occurred more often among patients with high blood pressure than among those who did not have high blood pressure. Therefore, depression is an important issue in the management of hypertension.

Although avoiding depression is understood to be an important goal in hypertension management, little attention has been paid to addressing this issue. Given that the burden of hypertension remains substantial in China, additional approaches to improving hypertension management are required. To bring about better hypertension management, it is essential to identify modifiable protective factors. Mental health has been shown to be influenced by individual-level factors such as socioeconomic status and social capital (SC) [7]. SC refers to “features of social organization such as networks, norms, and social trust that facilitate coordination and cooperation for mutual benefit.” Individuals can acquire individual-level SC, which may be utilized to pursue personal goals. Studies have also shown an inverse association between SC and depression among hypertensive patients [8]. Moreover, comorbid depression among people with hypertension may be linked to a lack of adherence to treatment, high complication rates, poor control of hypertension, and worse perceptions of quality of life [9]. However, to our knowledge, few studies have examined the associations between SC, depression, and perceived quality of hypertensive care in China.

In this study, we aimed to estimate the prevalence of symptoms of depression among patients with hypertension in PC settings. Additionally, we tried to identify the potential role of SC in the occurrence of symptoms of depression among patients with hypertension. The influence of depression on perceived quality of hypertensive care in PC settings was also examined. Our study findings can provide clues to policy makers and researchers to help better manage hypertension in PC in China.

## Methods

### Study design and sample

A cross-sectional study was carried out in Shenzhen, China. Using multistage cluster random sampling methods, we selected community health centers (CHCs; PC facilities) as the study setting. First, using this method, one of the 10 districts in Shenzhen—Longhua District, which includes six sub-districts—was selected. We then obtained a list of the CHCs in each sub-district from the Health Bureau of Longhua District. This was followed by selecting two CHCs from each sub-district using simple random sampling. Ultimately, we selected 12 CHCs for the study.

### Survey procedures

On-site surveys were conducted at the selected CHCs. The PC user population at these CHCs was used as the sampling frame. Using systematic sampling, we selected every fifth care user listed according to their entry order. The following inclusion criteria were applied in this selection: (1) aged > 18 years; (2) capable of communicating in Mandarin or Cantonese and providing informed consent; and (3) having resided in Shenzhen for more than 6 months. It was determined whether the participants had been professionally diagnosed with hypertension. Each potential participant’s blood pressure was also measured. To be selected for the study, patients had to have (1) a previous diagnosis of hypertension by a health care professional or (2) elevated blood pressure (≥ 140/90 mmHg). In each CHC, 100 hypertensive patients were selected, yielding a total sample of 1200. The data were collected via a face-to-face interview survey carried out by trained interviewers from March to September 2017. Assurances of anonymity and confidentiality were given to the participants following the acquisition of informed consent. A total of 1046 respondents completed the survey (response rate = 87.2%).

### Variables

#### Depressive symptoms

The World Health Organization-Five Well-Being Index was used to measure depression among the study participants. This index comprises the following five items, which respondents are instructed to assess in terms of perceived frequency in the last two weeks: (1) “I have felt cheerful and in good spirits”; (2) “I have felt calm and relaxed”; (3) “I have felt active and vigorous”; (4) “I woke up feeling fresh and rested”; and (5) “My daily life has been filled with things that interest me.” These items were answered on a six-point scale ranging from 0 (*strongly disagree*) to 5 (*strongly agree*). The total raw score was computed by summing the individual scores; the raw score thus had a theoretical range of 0 (absence of well-being) to 25 (maximal well-being). To facilitate comparison with prior studies, these raw scores were converted to a percentage scale ranging from 0 to 100 by multiplying the raw scores by four. Both international and national studies have demonstrated that this index is a valid method of screening for depression [10]. A score of less than 50 was defined as having depressive symptoms.

#### Social capital

The measurement of social capital was carried out at the individual level by assessing both structural and cognitive factors. The assessment of structural SC concerned formal networks and social ties. To measure formal networks, the respondents were asked, “Are you involved in any of these kinds of organizations?” The respondents answered separately for the following organization types: sports teams, political parties, associations (religious, technology, or volunteer organizations; associations of coworkers; hobby groups; community members’ groups; classmates’ associations; and family groups) (0 = *no*, 1 = *yes*). An unweighted sum score was then calculated (range: 0–10). Social ties were measured using the following questions: (1) “How often do you contact your family members or relatives?”; (2) “How often do you contact your friends?”; and (3) “How often do you contact your neighbors?” The response options were 1 = *never*, 2 = *seldom*, 3 = *sometimes*, 4 = *frequently*, and 5 = *always*. We calculated an unweighted sum score that ranged from 3 to 15. Trust (reflecting cognitive SC) was measured using the following five items: “Generally speaking, would you say that (1) family members or relatives, (2) friends, (3) neighbors, (4) primary care providers, or (5) hospital doctors can be trusted?” These items were assessed on a five-point response scale.

#### Perceived quality of care

Data on perceived quality of care were collected using the adapted and validated Chinese version of the Primary Care Assessment Tool. Specifically, respondents were asked about 10 PC attributes, including continuity, first contact accessibility and utilization, comprehensiveness of service availability and provision, coordination of services and information, community orientation and cultural competence, and family centeredness. These items have been used to monitor PC systems. Scoring was done on a four-point Likert-type scale, where high scores indicated higher quality. Perceptions related to each of these attributes was represented by a mean score for all the questions under the subject heading (ranging from 1 to 4). A total score reflecting perceptions of the overall PC quality was calculated by summing the individual scores for each attribute (range: 10–40).

#### Covariates

The included covariates were age group (18–44 years, 45–60 years, or > 60 years), gender (male or female), employment status (employed or unemployed), level of education (primary school or lower, middle school, high school or equivalent, or 3-year college or higher), insurance status (covered by local or non-local health insurance schemes or unknown), migration status (locals or migrants based on household registration [*hukou*]), duration since diagnosis of hypertension, and family history of hypertension (yes, no, or unknown).

### Statistical analyses

All analyses were conducted using SPSS, Version 20.0. Descriptive statistics were calculated for the following respondent characteristics: gender, age group, employment status, level of education, migration status, insurance status, family history of hypertension, and duration since hypertension diagnosis. The prevalence of depressive symptoms was also calculated. The independent two-sample *t*-test was used to compare SC and perceived quality of care between the respondents with depressive symptoms and those who did not have depressive symptoms. Multiple logistic regression models were used to test the associations between the different types of SC and depression, and multiple linear regression models were used to test the association between depression and perceived quality of care. The models were adjusted for gender, age group, employment status, level of education, migration status, insurance status, family history of hypertension, and duration since hypertension diagnosis. Odds ratios [ORs] and *β* coefficients (along with 95% confidence intervals) are reported. All *P*-values < 0.05 were considered to indicate a statistically significant relationship.

## Results

The participants for whom no information on gender was available were excluded from the final analysis to avoid introducing bias. Ultimately, a total of 977 participants were included in the final analysis. The participants had a mean age of 55.5 years. Approximately one-third (27.9%) had a primary school education or below, whereas only 12.0% had a 3-year college education or higher. More than half of the participants were employed. The proportions of participants covered by local and non-local health insurance schemes were almost equal. Most of the participants were migrants (85.6%). About half of the participants had a family history of hypertension. The mean duration since hypertension diagnosis was 6.1 years. (Table 1).

**Table 1:** Socio-demographic characteristics of the participants

Characteristics	No. of total participants (%)
Age (mean, SD)	55.5 (11.4)
18–44	156 (16.7)
45–60	451 (48.2)
> 60	329 (35.1)
Gender	
Male	584 (59.8)
Female	393 (40.2)
Education	
Primary school or below	268 (27.9)
Middle school	332 (34.6)
High school or equivalent	244 (25.4)
3-year college or above	115 (12.0)
Occupation	
Employed	521 (54.6)
Unemployed	433 (45.4)
Insurance	
Non-local insurance	413 (44.3)
Local insurance	416 (44.6)
Unknown	103 (11.1)
Registration	
Locals	132 (14.4)
Migrants	784 (85.6)
Family history of hypertension	
Yes	432 (46.3)
No	351 (37.6)
Unknown	150 (16.1)
Year of hypertension (mean, SD)	6.1 (6.0)

*SD* standard deviation

Mean WHO-5 score reported by the participants was 72.4. In total, 10.7% of the participants had depressive symptoms. The reported mean scores for network, social ties and trust were 2.09, 10.56 and 3.84, respectively. The mean score for perceived quality of care was 32.3 (Table 2).

**Table 2:** Scores of WHO-5, social capital, quality of care reported by the participants

Variables	Mean (SD) of total participants
WHO-5 score	72.4 (18.82)
Depression	
Yes (No. %)	104 (10.7)
Social capital	
Network	2.09 (2.03)
Social ties	10.56 (2.07)
Trust	3.84 (0.65)
Quality of care	32.3 (3.48)

*SD* standard deviation

Table 3 shows that the participants without depressive symptoms had higher scores on participation in formal organizations (1.55 vs. 2.17, *P* = 0.002), social ties (9.63 vs. 10.67, *P* < 0.001), and ability to trust others (3.46 vs. 3.89, *P* < 0.001), compared with their counterparts with depressive symptoms. After adjusting for socio-demographic characteristics using multiple logistic regression (Table 4), the statistically significant differences remained for social ties (OR = 1.314, 95% CI 1.165–1.483; *P* < 0.001) and trust (OR = 2.535, 95% CI 1.741–3.691; *P* < 0.001). Participants with local health insurance coverage were less likely to have depressive symptoms than those covered by non-local insurance schemes (OR = 0.392, 95% CI 0.188–0.819; *P* < 0.05) (Table 4).

**Table 3:** Univariate analysis of the associations between social capital, quality of care and depression

Variables	Depression, mean (SD)		*P*^a^
	Yes	No	
Social capital			
Network	1.55 (0.18)	2.17 (0.07)	0.002
Social ties	9.63 (0.19)	10.67 (0.07)	< 0.001
Trust	3.46 (0.07)	3.89 (0.02)	< 0.001
Quality of care	30.5 (3.53)	32.5 (3.41)	< 0.001

^a^Independent two-sample *t*-tests

SD: standard deviation

**Table 4:** Multiple logistic regression analysis showing the associations between social capital and depression

Characteristics	Depression, OR (95% CI)^a^
Age	
18–44	1
45–60	1.217 (0.587, 2.520)
> 60	1.912 (0.708, 5.166)
Gender	
Male	1
Female	0.993 (0.536, 1.842)
Education	
Primary school or below	1
Middle school	1.347 (0.649, 2.798)
High school or equivalent	0.869 (0.383, 1.969)
3-year college or above	1.202 (0.410, 3.518)
Occupation	
Employed	1
Unemployed	0.567 (0.256, 1.256)
Insurance	
Non-local insurance	1
Local insurance	0.392 (0.188, 0.819)*
Unknown	0.339 (0.148, 0.777)*
Registration	
Locals	1
Migrants	0.394 (0.145, 1.073)
Family history of hypertension	
Yes	1
No	1.705 (0.940, 3.092)
Unknown	1.871 (0.814, 4.300)
Year of hypertension	0.993 (0.949, 1.039)
Social capital	
Network	1.071 (0.928,1.236)
Social ties	1.314 (1.165,1.483)***
Trust	2.535 (1.741, 3.691)**

*OR* odds ratio, *CI* confidence interval

^*^P < 0.05; **P < 0.01; ***P < 0.001

^a^Multiple logistic regression models with depression as the dependent variable, adjusted for socio-demographic factors including age group, gender, level of education, migration status, insurance status, employment status, duration since hypertension diagnosis, and family history of hypertension

We also conducted a sensitivity analysis by simultaneously including formal network, social ties, and trust scores in the multiple logistic regression model. In this model, social ties (OR = 1.202, 95% CI 1.039–1.390; *P* = 0.014) and trust (OR = 2.055, 95% CI 1.339–3.156; *P* = 0.001) were significantly associated with the prevalence of depressive symptoms (data not shown).

Participants without depressive symptoms had higher Primary Care Assessment Tool scores than did those with depressive symptoms (30.5 vs. 32.5, *P* < 0.001). (Table 3) Following adjustment for the confounders, significant variations between those with and without depressive symptoms remained (*β* = 1.341, 95% CI 0.463–2.219; *P* = 0.003) (Table 5). It was also found that participants with local health insurance scheme coverage had better perceived quality of care when compared with those without (*β* =  − 0.492, 95% CI − 0.920 to − 0.064; *P* < 0.05). (Table 5).

**Table 5:** Multiple linear regression analysis showing the associations between depression and perceived quality of care

Characteristics	Quality of care, *β* (95% CI)^a^
Age	− 0.013 (− 0.045, 0.019)
Gender	
Male	0
Female	0.176 (− 0.420, 0.771)
Education	
Primary school or below	0
Middle school	0.041 (− 0.658, 0.739)
High school or equivalent	0.640 (− 0.127, 1.407)
3-year college or above	0.439 (− 0.591, 1.469)
Occupation	
Employed	0
Unemployed	− 0.295 (− 1.028, 0.438)
Insurance	
Local insurance	0
Non-local insurance	− 0.492 (− 0.920, − 0.064)*
Unknown	0.352 (− 0.215, 0.462)
Registration	
Locals	0
Migrants	− 0.053 (− 0.834, 0.728)
Family history of hypertension	
Yes	0
No	0.316 (− 0.216, 0.893)
Unknown	0.004 (− 0.777,0.784)
Year of hypertension	− 0.036 (− 0.088, 0.016)
Depression	
Yes	1.341 (0.463,2.219)**
No	0

^a^Multiple linear regression model with perceived quality of care as the dependent variable, adjusted for socio-demographic factors including age group, gender, level of education, migration status, insurance status, employment status, duration since hypertension diagnosis, and family history of hypertension

*CI* confidence interval

^*^P < 0.05; **P < 0.01

## Discussion

Our study investigated depressive symptoms in a large-scale representative sample of patients with hypertension who attended PC centers in Shenzhen, China. We also examined the links between various types of SC and depression. The influence of depressive symptoms on perceived quality of care was also investigated. Depression was found to be an important public health concern. In the PC facilities, 10.7% of the participants with hypertension had symptoms of depression. Two components of SC (social ties and trust) were identified as protective factors for depression among patients with hypertension in PC settings. Our study indicated that depressive symptoms were negatively linked with perceived care quality.

Our finding of depressive symptoms among 10.7% of participants with hypertension who attended the selected PC facilities is lower than estimates reported in other national and international studies. A cross-sectional survey conducted by Mahmood et al. [11] in Karachi, Pakistan, indicated that 40.1% of patients with hypertension suffered from depression. In Nigeria, 26.7% of patients with hypertension were found to suffer from depression [12]. A meta-analytic study involving 41 studies showed that, in China, 28.5% of patients with hypertension had depression and indicated that the prevalence rates in clinical samples are higher than those estimated by community studies [13]. However, in 2015, the World Health Organization reported that, globally, only 4.4% of hypertensive patients experienced depression [14]. This World Health Organization estimate is substantially lower that what we found in this study. However, when compared with findings in the general public in Shenzhen, the estimate of the prevalence of hypertension in our study is higher [15]. Newale and Bachani [16] have suggested that patients with depression often suffer from comorbidities such as hypertension and diabetes, also noting that people suffering from depression had nearly three times as many chronic medical conditions compared with people who did not have depression.

Our study indicated that two types of SC (social ties and trust) are negatively associated with the occurrence of depressive symptoms in hypertensive patients attending PC facilities. Our finding of an inverse association between depression and social ties is consistent with previous studies. It is well known that social ties can benefit mental health. Kawachi and Berkman [17] also demonstrated that social ties benefit mental health. In China, Liu et al. [18] showed that social ties with neighbors enhance migrants’ subjective well-being in a direct manner. This finding is reasonable because close social ties can prevent individuals from becoming isolated and help to make communication easier, which promotes the transmission of positive health messages.

Our finding of an inverse association between social trust and the occurrence of depressive symptoms is also consistent with previous observations in both developed and developing countries. In Korea, Han et al. [19] demonstrated that trust was significantly associated with depressive symptoms among older adults in a nationally representative sample of the population. A cross-sectional study conducted by Forsman et al. [20] also found that low levels of trust were associated with depressive symptoms among the Swedish and Finnish populations. In China, Zhou et al. [21] reported a negative association between depressive symptoms and social trust among primiparas. This observation was in line with Cao et al.’s [22] finding that level of trust was inversely correlated with depression in the Chinese population. The stress-buffering model and the main-effect model are two hypotheses that may help to explain the mechanism of the association between trust with depression [19]. Our study findings are promising and suggest a significant protective effect of some aspects of SC on depressive symptoms among hypertensive patients in PC settings.

Our results showed that depression is negatively associated with the perception of the quality of hypertensive care received by patients in PC facilities. This finding is consistent with previous studies. A study conducted by Benjamin et al. [23] showed that, compared with people without depression, people with depression had statistically significantly more problems related to the accessibility, continuity, coordination, and comprehensiveness of PC services. Leonard et al. [24] demonstrated a link between depression and decreased quality of care among adults with diabetes. Our findings imply that addressing hypertension alone may not be sufficient to improve medical outcomes, given the high prevalence of depression in patients with hypertension. Symptoms of depression such as low motivation and hopelessness may interfere with these patients’ ability to obtain and maintain treatment. Treating depressive symptoms may therefore play a major role in the improvement of PC for patients who suffer from both hypertension and depression. Strategies addressing both depression and other comorbidities should be preferred.

Results showed that participants covered by local health insurance schemes were less likely to have depressive symptoms than those covered non-local health insurance schemes. Results also showed that participants with local health insurance coverage reported better perceived quality of care when compared with those with non-local health insurance coverage. Our findings are consistent with those of previous studies. Rivera-Hernandez and Galarraga [25] found that uninsured people were less likely to receive screening services. The study by Hendriks et al. [26]further showed that lack of insurance decreased access to health care. This might be one possible explanation of the higher prevalence rate of depression among hypertensive patients with non-local health insurance coverage. A study by Bleich et al. [27] also demonstrated that health insurance was associated with improved quality of hypertensive care. The study by Hendriks et al. [26] confirmed the findings of Bleich et al. [27].

Our study has several limitations. First, the study population was site-based, which limits the generalizability of the study findings. Second, the information regarding perceived quality of care and depressive symptoms was self-reported by patients, which may have introduced recall bias. However, we carefully defined our survey questions, and the interviews were conducted by well-trained interviewers. Third, because this was a cross-sectional study, we could not establish causal inference between SC, depression, and perceived quality of care. Further longitudinal or interventional studies are warranted to enhance our understanding of the issues investigated in this study. Fourth, caution is warranted when comparing the findings of our study with those of previous studies because of differences in the definition and measurement of SC.

## Conclusions

In conclusion, we found a high occurrence of symptoms of depression among patients with hypertension in PC settings, as well as an inverse association between depressive symptoms and perceived quality of care. Our findings suggest that strategies to address hypertension together with comorbidities (e.g., depressive symptoms) should be used to improve the management of hypertension. Because of the association between antihypertensive medications and depression, patients receiving antihypertensive medications should be periodically assessed for depression. If this medication plays a role in depression among these patients, alternatives with lower risk should be used. We also found an inverse association between SC and the occurrence of depressive symptoms, which implies that social interventions should be designed and implemented to improve the mental health of hypertensive patients, although causal inference was not established by the current study.

## Data Availability

All relevant data were presented in the manuscript.

## Abbreviations

PC: Primary care
SC: Social capital
CHCs: Community health centers
OR: Odds ratio
CI: Confidence interval
